# Patient-reported outcomes one year after positive sentinel lymph node biopsy with or without axillary lymph node dissection in the randomized SENOMAC trial

**DOI:** 10.1016/j.breast.2022.02.013

**Published:** 2022-03-01

**Authors:** Matilda Appelgren, Helena Sackey, Yvonne Wengström, Karin Johansson, Johan Ahlgren, Yvette Andersson, Leif Bergkvist, Jan Frisell, Dan Lundstedt, Lisa Rydén, Malin Sund, Sara Alkner, Birgitte Vrou Offersen, Tove Filtenborg Tvedskov, Peer Christiansen, Jana de Boniface, the SENOMAC Trialists' Group

**Affiliations:** a Department of Molecular Medicine and Surgery, Karolinska Institutet, 171 76, Stockholm, Sweden; b Division of Cancer, Department of Breast, Endocrine Tumors and Sarcoma, Karolinska University Hospital, 171 64, Solna, Sweden; c Karolinska Comprehensive Cancer Center, Karolinska University Hospital, 171 64, Solna, Sweden; d Department of Neurobiology, Care Sciences and Society, Division of Nursing, Karolinska Institutet, 141 52, Huddinge, Sweden; e Department of Health Sciences, Lund University, 221 00, Lund, Sweden; f Department of Oncology, University Hospital, 701 85, Örebro, Sweden; g Regional Oncology Centre, Mid-Sweden Health Care Region, 751 85, Uppsala, Sweden; h Department of Surgery, Västmanland County Hospital, 721 89, Västerås, Sweden; i Västmanland County Hospital, Center for Clinical Research, Uppsala University, 721 89, Västerås, Sweden; j Department of Oncology, Institute of Clinical Sciences, Sahlgrenska Academy, University of Gothenburg, Sahlgrenska University Hospital, 413 45, Gothenburg, Sweden; k Division of Surgery, Department of Clinical Sciences Lund, Lund University, 221 84, Lund, Sweden; l Department of Surgery and Gastroenterology, Skåne University Hospital, 214 28, Malmö, Sweden; m Department of Surgical and Perioperative Science/Surgery, Umeå University, 709 87, Umeå, Sweden; n Department of Surgery, University of Helsinki and Helsinki University Hospital, PO Box 440, Helsinki, Finland; o Division of Oncology, Department of Clinical Sciences Lund, Skåne University Hospital, Lund University, 221 84, Lund, Sweden; p Department of Experimental Clinical Oncology Aarhus University Hospital, 8200, Aarhus, Denmark; q Department of Oncology, Aarhus University Hospital, 8200, Aarhus, Denmark; r Danish Breast Cancer Group Center and Clinic for Late Effects, Aarhus University Hospital, 8200, Aarhus, Denmark; s Department of Breast Surgery, Rigshospitalet, 2100, Copenhagen, Denmark; t Department of Plastic and Breast Surgery, Aarhus University Hospital, 8200, Aarhus, Denmark; u Department of Surgery, Capio St Göran's Hospital, 112 19, Stockholm, Sweden

**Keywords:** Breast cancer, Patient-reported outcome measures, Health-related quality of life, Arm morbidity, Sentinel lymph node biopsy, Axillary lymph node dissection, ALND, Axillary lymph node dissection, BCS, Breast-conserving surgery, CTV, Clinical target volume, HRQoL, Health-related quality of life, PROM, Patient-reported outcome measure, RT, Radiotherapy, SLN, Sentinel lymph node, SLNB, Sentinel lymph node biopsy

## Abstract

**Introduction:**

This report evaluates whether health related quality of life (HRQoL) and patient-reported arm morbidity one year after axillary surgery are affected by the omission of axillary lymph node dissection (ALND).

**Methods:**

The ongoing international non-inferiority SENOMAC trial randomizes clinically node-negative breast cancer patients (T1-T3) with 1–2 sentinel lymph node (SLN) macrometastases to completion ALND or no further axillary surgery. For this analysis, the first 1181 patients enrolled in Sweden and Denmark between March 2015, and June 2019, were eligible. Data extraction from the trial database was on November 2020. This report covers the secondary outcomes of the SENOMAC trial: HRQoL and patient-reported arm morbidity. The EORTC QLQ-C30, EORTC QLQ-BR23 and Lymph-ICF questionnaires were completed in the early postoperative phase and at one-year follow-up. Adjusted one-year mean scores and mean differences between the groups are presented corrected for multiple testing.

**Results:**

Overall, 976 questionnaires (501 in the SLN biopsy only group and 475 in the completion ALND group) were analysed, corresponding to a response rate of 82.6%. No significant group differences in overall HRQoL were identified. Participants receiving SLN biopsy only, reported significantly lower symptom scores on the EORTC subscales of pain, arm symptoms and breast symptoms. The Lymph-ICF domain scores of physical function, mental function and mobility activities were significantly in favour of the SLN biopsy only group.

**Conclusion:**

One year after surgery, arm morbidity is significantly worse affected by ALND than by SLN biopsy only. The results underline the importance of ongoing attempts to safely de-escalate axillary surgery.

**Trial registration:**

The trial was registered at clinicaltrials.gov prior to initiation (https://clinicaltrials.gov/ct2/show/NCT 02240472).

## Highlights

• Omission of ALND significantly reduces patient-reported arm morbidity.
• SLNB versus ALND results in significant less pain and better physical function.
• HRQoL is not affected by de-escalated axillary surgery.
• Complaints from axillary surgery are evaluated with patient-reported outcomes.

## Graphical abstract

**Figure:**
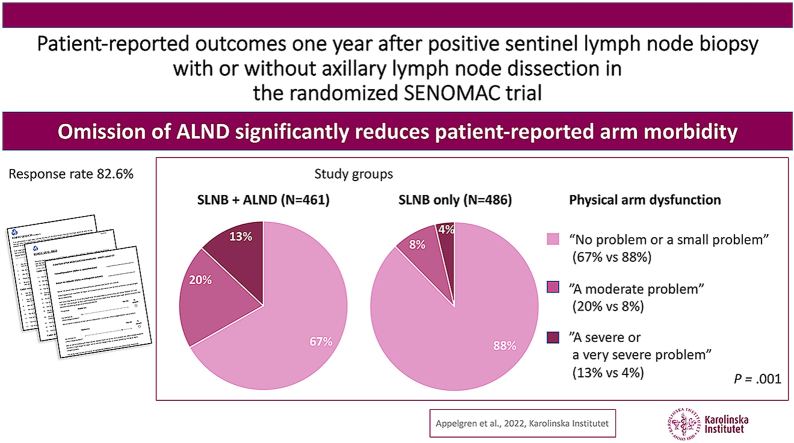

## 1 Introduction

The driving force behind current efforts to de-escalate axillary staging surgery in breast cancer is the search for a balance between oncological safety and the preservation of arm function and health-related quality of life (HRQoL). Since the beginning of this century, it was clarified that the omission of axillary lymph node dissection (ALND) after a negative sentinel lymph node biopsy (SLNB) is oncologically safe [[1], [2], [3]]. Subsequently, randomized studies have indicated that ALND does not improve survival or locoregional control in patients with sentinel lymph node (SLN) micrometastases or 1–2 SLN macrometastases undergoing breast-conserving surgery [4,5]. The ongoing randomized SENOMAC trial aims to both validate and extend those findings [6]. Randomized data on patient-reported outcome measures (PROMs) regarding arm morbidity and HRQoL are scarce. Even though the randomized AMAROS and OTOASOR trials, showing non-inferior outcomes in patients with SLN micro- or macrometastases who received axillary radiotherapy (RT) instead of completion ALND, integrated PROMs, detailed results have not been published. Instead, it was briefly stated that no differences were observed between the groups [7,8]. The incidence of lymphoedema, however, was twice as high after ALND than after axillary RT in the AMAROS trial [7].

Arm morbidity is a common consequence of ALND and may consist of arm swelling [[7], [8], [9], [10], [11], [12]], numbness [4,8,9,13,14], impaired shoulder movement, and pain [8], limiting physical activity [10,15] and may delay return to work [[16], [17], [18]]. Arm morbidity may be evaluated by PROMs or by objective measurements, but importantly, these do not necessarily align [12,19,20]. Sackey et al. reported that patient-reported symptoms of lymphoedema were associated with loss of HRQoL while objectively measured lymphoedema was not [19]. From a patient perspective, the evaluation of PROMs should therefore be an integral part of trials on de-escalation of axillary surgery. Here, we present one-year PROM data from the randomized SENOMAC trial.

## 2 Materials and methods

### 2.1 Study design

The ongoing SENOMAC trial, initiated in 2015, is an international non-inferiority trial including clinically node-negative adult, breast cancer patients (T1-T3) with 1–2 SLN macrometastases who are randomized 1:1 to ALND or no further axillary surgery. As an extension to previous trials, patients with T3 tumours and those treated with mastectomy are also eligible. The primary outcome is overall survival; HRQoL and patient-reported arm morbidity are among the secondary outcomes. The SENOMAC protocol has been published in detail elsewhere [6].

**Fig. 1:**
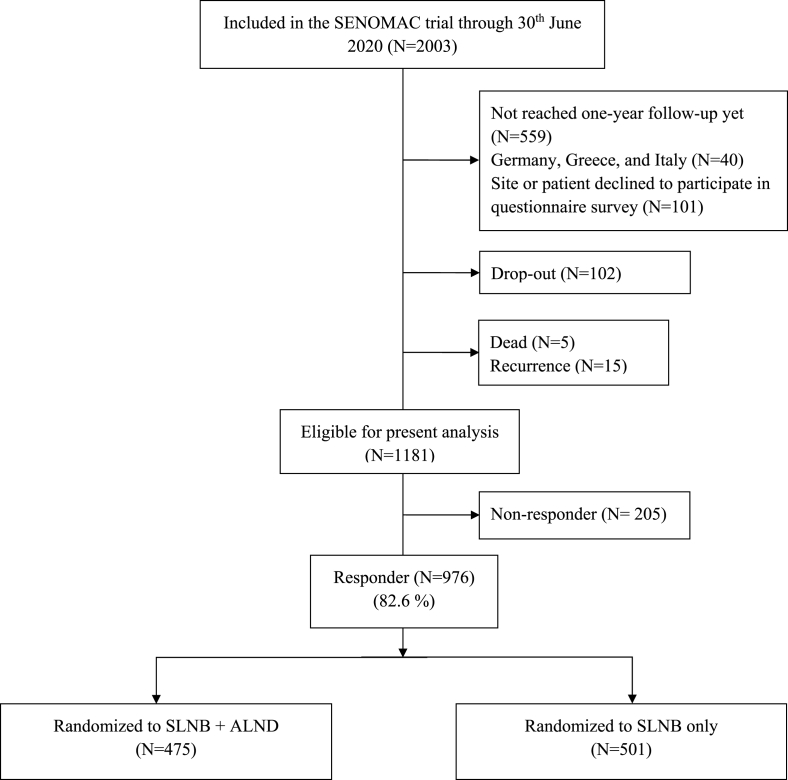
Trial Consort. Number of patients in the present analysis. Drop-outs include withdrawal of consent, erroneous enrolment, termination due to physician's decision, and loss to follow-up.

Participants included in this analysis had reached their one-year follow-up by June 30, 2020 and had responded to all questionnaires at least once. Included participants were treated at 33 hospitals in Sweden (N = 733, first patient on March 9, 2015) and Denmark (N = 243, first patient on April 20, 2017). The eligible number of participants from German, Italian, and Greek sites, which were initiated subsequently, were too small to be included in the present analysis (N = 22, 5, and 9 per country, respectively). Clinical and follow-up data were extracted from the trial database on November 1, 2020 and linked to corresponding PROM data separately registered in an online database. Patients experiencing a recurrence before the one-year follow-up date, those not understanding any of the languages provided in the questionnaires, or who declined to receive questionnaires were excluded (Fig. 1).

In SENOMAC, informed consent is either obtained prior to SLNB in those planned for frozen section or at the first postoperative visit, when final histopathology is available. The first questionnaire was distributed as an early postoperative measurement once inclusion criteria were confirmed, i.e. after SLNB. Half of the patients whose eligibility was confirmed by frozen section and who were thus randomized during surgery had already undergone a completion ALND by the time of first questionnaire completion. Therefore, the early postoperative measurement reflects different extents of axillary surgery.

Questionnaires were distributed once again after one year. Questionnaires completed later than four months after the enrolment date, or outside of the time frame of two months prior or four months after the corresponding one-year follow-up date, were not included in the analysis.

According to Danish Breast Cancer Group guidelines, the RT axillary clinical target volume (CTV) also includes axillary level 1 in addition to levels 2–3 when less than nine lymph nodes are removed, which includes all patients undergoing SLNB only. In Sweden, however, axillary CTV is not dependent on the number of lymph nodes removed even though the inclusion of axillary level 1 may vary among sites. Consequently, it is expected that the SLNB only group received RT to axillary level 1 more often than the SLNB + ALND group.

Written informed consent was obtained from all participants. Ethical permission was granted by the Regional Ethical Review Board in Stockholm, Sweden, in 2014 (2014/1165-31/1) and by the Regional Ethical Review Board in Viborg, Denmark, in 2015 (1-10-72-284-15).

### 2.2 Questionnaires

The European Organization for Research and Treatment of Cancer (EORTC) QLQ-C30 (version 3.0), QLQ-BR23, and the Lymphedema Functioning Disability and Health (Lymph-ICF) electronic- or paper-based questionnaires were used to assess HRQoL and arm morbidity.

The EORTC QLQ-C30 questionnaire measures HRQoL among cancer patients in general and consists of 30 items divided into multi-item scales and single items. The multi-item scales include one global health and quality of life (QoL) scale, five function subscales (physical, role, emotional, cognitive, social) and three symptom subscales (fatigue, nausea and vomiting, pain). The single items are dyspnoea, insomnia, appetite loss, constipation, diarrhoea, financial difficulties [21].

The EORTC QLQ-BR23 questionnaire measures functions and symptoms related to breast cancer treatment and consists of 23 items divided into two functional subscales (body image and sexuality) and three symptom subscales (systemic therapy side effects, arm symptoms, breast symptoms) and three single items (sexual enjoyment, upset by hair loss, future perspective) [22].

In both questionnaires, the functional and symptom subscales as well as the single items correspond to a response scale 1–4 (not at all, a little, quite a bit, very much) while global health and QoL correspond to a response scale 1–7 (very poor to excellent). Each scale produces a total score from 0 to 100. High scores on global health and QoL represent a better HRQoL, high scores on functional subscales indicate better function, and high scores on symptom subscales indicate more severe problems [23]. The multi-item scale “global health and QoL” will be termed HRQoL in the following text. The questionnaires EORTC QLQ-C30 and QLQ-BR23 have been developed and tested for reliability and validity by the EORTC group [21,22]. Validated Swedish and Danish translations were downloaded from www.eortc.org with permission for academic use.

The Lymph-ICF questionnaire has been developed and validated to assess arm-related impairments in function, activity limitations, and participation restrictions in patients with breast cancer-related lymphedema [24]. Since SENOMAC does not focus on prevalent lymphedema, the introduction was adapted. The questionnaire consists of 29 items divided into five domains: physical function, mental function, household activities, mobility activities, and life and social activities and produces an overall domain, termed “Lymph-ICF total”. Each item is scored on a visual analogue scale (0–100 mm) resulting in domain scores ranging from 0 to 100. Higher scores indicate more severe arm dysfunction. Lymph-ICF scores also categorize into “no problem”, “a small problem”, “a moderate problem”, “a severe problem”, and “a very severe problem” [24]. By the time of initiation of the SENOMAC trial, there was no Swedish translation of the Lymph-ICF. Translation was performed according to the Swedish version of International Classification of Functioning, Disability and Health (ICF) [25] after permission from the author of the Lymph-ICF. The internal consistency of the Swedish translation was tested within the SENOMAC trial, with a Cronbach's alpha of 0.96, ranging from 0.86 to 0.93 of each domain. The Danish version of the Lymph-ICF has been validated and tested for reliability [26].

### 2.3 Statistical analysis

The main objective of the present analysis was to compare HRQoL and patient-reported arm morbidity one year after surgery between the two randomization groups. Scores of EORTC QLQ-C30, QLQ-BR23 and Lymph-ICF were calculated using the questionnaire-specific scoring manuals [23,24]. All analyses were based on complete cases. Descriptive statistics are presented as numbers and percentages (%), and as median values with their ranges (min-max). When testing differences between randomization groups and between survey responders and non-responders, two-sided Chi-square tests, Fisher's exact tests and independent t-tests, were used as appropriate.

**Fig. 2:**
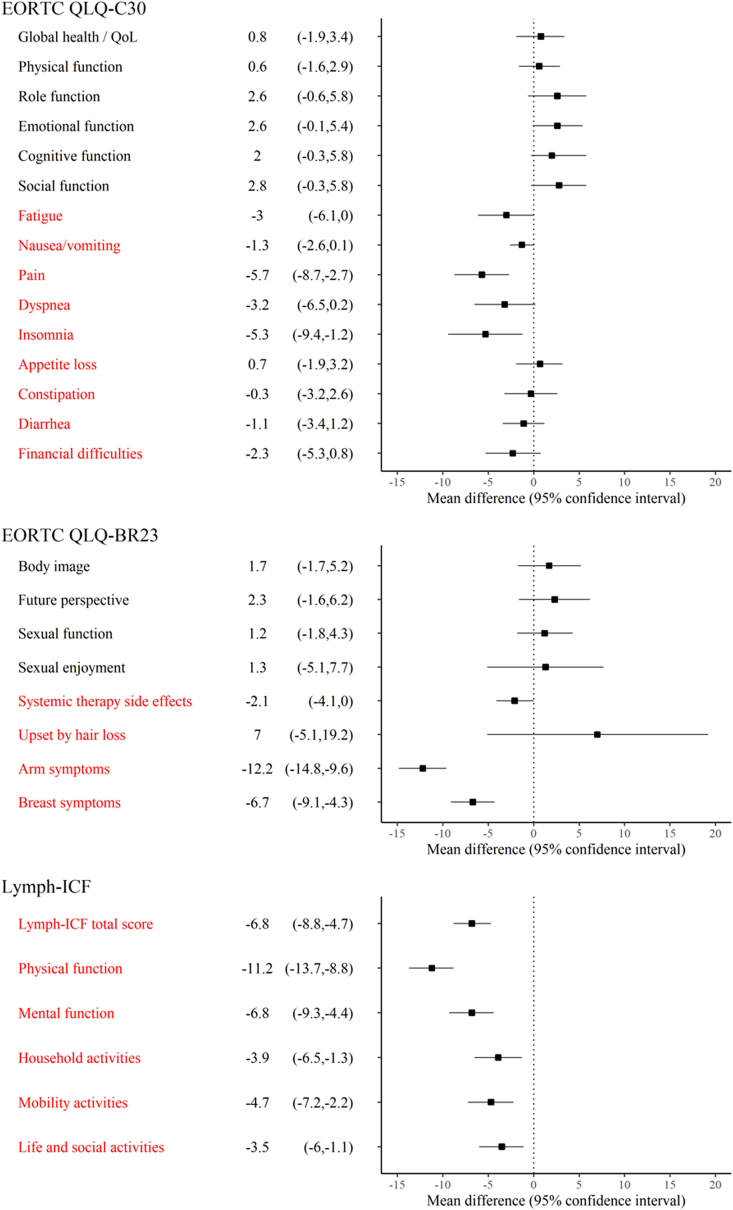
Adjusted mean differences (95% CI) between randomization groups at one-year follow-up for each subscale of the EORTC questionnaires and domains of the Lymph-ICF questionnaire with the SLNB + ALND group as a reference. Positive mean differences of black texted items indicate better function, negative mean differences of red texted items indicate less symptoms or less impaired function in comparison with reference.

Since randomization was stratified by country and an unequal distribution of axillary CTVs was anticipated, all questionnaire mean scores were adjusted for country and for type of received axillary RT (none, including level 1, not including level 1) by two-way factorial ANOVA analysis. Ordinal logistic regression model was used for categorized Lymph-ICF results. A test of the proportional odds assumption was assessed for all ordinal logistic regression models. If the assumption was violated, a nominal regression model was performed, using the lowest outcome category (“No or small problem”) as reference. Adjusted means from ANOVA models and odds ratios from ordinal logistic regression are presented with their corresponding 95% confidence interval (CI). ANOVA F test and Wald test, respectively, were used analysing the mean score and categorized result differences between the randomization groups. Furthermore, adjusted mean differences with 95% CI from the ANOVA models are presented in a forest plot for better visualization (Fig. 2). To account for multiple testing, the significance threshold was adjusted by Bonferroni correction which resulted in a required two-sided significance level below 0.0017 for questionnaire mean scores, and below 0.0087 for categorized Lymph-ICF results.

All analyses used SPSS statistical software version 27 (IBM Corp., Armonk, NY, USA).

## 3 Results

Overall, 976 out of 1181 eligible patients were included in the present analysis: 475 patients in the SLNB + ALND group and 501 patients in the SLNB only group, corresponding to a response rate of 82.6% (Fig. 1). The overall drop-out rate was 7.8% (N = 102). Drop-out due to withdrawal of consent was more common in the SLNB + ALND group (N = 51, 3.9%) than in the SLNB only group (N = 7, 0.5%; *P* < .001). Response rates of individual items ranged between 94% and 97% except for sexual enjoyment (32%) and upset by hair loss (16%). Patient and treatment characteristics are presented in Table 1.

**Table 1:** Patient and treatment characteristics per randomization group.

	SLNB + ALND (N = 475)	SLNB only (N = 501)
**Type of breast surgery**		
BCS	314 (66.1)	331 (66.1)
Mastectomy	161 (33.9)	170 (33.9)
**No. of lymph nodes removed,** median (range)	14 (1–50)	2 (1–15)
Missing	4	1
**Age,** median (range)	61 (34–87)	62 (23–92)
<50 years	85 (17.9)	90 (18.0)
50–65 years	214 (45.1)	205 (40.9)
>65 years	176 (37.1)	206 (41.1)
**Country**		
Sweden	361 (76.0)	372 (74.3)
Denmark	114 (24.0)	129 (25.7)
Chemotherapy*		
Yes	329 (69.3)	329 (65.7)
No	146 (30.7)	172 (34.3)
HER 2 targeted therapy**		
Yes	52 (10.9)	58 (11.6)
No	423 (89.1)	443 (88.4)
Endocrine therapy**		
Yes	431 (90.7)	465 (92.8)
No	43 (9.1)	36 (7.2)
Missing	1 (0.2)	
**Radiotherapy**		
Breast/chest wall and regional lymph nodes	448 (94.3)	470 (93.8)
Breast/chest wall only	14 (2.9)	20 (4.0)
None	12 (2.5)	11 (2.2)
Missing	1 (0.2)	

Presented as numbers and percentages if not stated otherwise. *Chemotherapy may be received before or after surgery. **Ongoing treatment at one-year follow-up. SLNB: sentinel lymph node biopsy, ALND: axillary lymph node dissection, BCS: breast-conserving surgery.

A non-responder analysis showed non-responders being significantly younger than responders (median age 58 (range 37–94) versus 62 (range 23–92) years, *P* = .005) as depicted in Supplementary Table 1.

### 3.1 EORTC QLQ-C30 and EORTC QLQ-BR23

One year after surgery, no significant differences were found between the randomization groups when evaluating HRQoL and function subscales. Participants operated with SLNB only however reported significantly less morbidity on the symptom subscales of pain, arm symptoms, and breast symptoms (Table 2 and Fig. 2). The delta value regarding HRQoL, i.e., the difference between early postoperative measurement and one-year follow-up among those participants who had completed questionnaires at both time points (N = 907), was 7.40 (95% CI: 5.40–9.41) in the SLNB + ALND group and 4.63 (95% CI:2.66–6.60) in the SLNB only group (*P* = .053), implying a significantly larger recovery of HRQoL in those individuals undergoing ALND.

**Table 2:** Adjusted EORTC QLQ-C30 and EORTC QLQ-BR23 mean function and symptom scores (95% CI), at early postoperative measurement and at one-year follow-up in the SLNB + ALND versus the SLNB only group.

	Early postoperative measurement				1-year follow-up			
	SLNB + ALND (N = 457)	SLNB only (N = 503)	Mean difference	P*	SLNB + ALND (N = 475)	SLNB only (N = 501)	Mean difference	P*
**EORTC QLQ-C30 version 3.0**								
Global health/QoL	65.0 (63.0, 67.1)	68.3 (66.3, 70.2)	3.2 (0.6, 5.9)	.017	73.8 (71.2, 76.4)	74.4 (72.0, 77.0)	0.8 (−1.9, 3.4)	.307
**Function subscales**								
Physical function	82.5 (80.9, 84.1)	82.9 (81.4, 84.4)	0.4 (−1.6, 2.5)	.684	83.6 (81.4, 85.7)	84.2 (82.1, 86.3)	0.6 (−1.6, 2.9)	.568
Role function	65.0 (62.1, 67.9)	70.5 (67.7, 73.2)	5.5 (1.7, 9.3)	.005	81.0 (77.9, 84.1)	83.6 (80.6, 86.5)	2.6 (−0.6, 5.8)	.108
Emotional function	70.5 (68.2, 72.8)	73.3 (71.1, 75.5)	2.8 (−0.1, 5.8)	.060	78.3 (75.6, 80.9)	80.9 (78.3, 83.5)	2.6 (−0.1, 5.4)	.061
Cognitive function	81.6 (79.5, 83.7)	82.8 (80.9, 84.8)	1.2 (−1.5, 3.9)	.373	80.4 (77.6, 83.1)	82.4 (79.7, 85.0)	2.0 (−0.8, 4.8)	.167
Social function	78.4 (76.1, 80.7)	81.6 (79.4, 83.8)	3.2 (0.2, 6.2)	.034	83.2 (80.2, 86.1)	85.9 (83.0, 88.8)	2.8 (−0.3, 5.8)	.076
**Symptom subscales/items**								
Fatigue	35.6 (33.3, 37.9)	33.3 (31.1, 35.5)	−2.3 (−5.3, 0.71)	.136	30.5 (27.6, 33.5)	27.5 (24.6, 30.3)	- 3.0 (−6.1, 0.0)	.052
Nausea/vomiting	5.7 (4.5, 6.9)	5.7 (4.6, 6.9)	0.0 (−1.5, 1.6)	.976	4.4 (3.1, 5.7)	3.2 (1.9, 4.4)	- 1.3 (−2.6, 0.1)	.072
Pain	30.3 (28.0, 32.7)	22.3 (20.0, 24.5)	−8.1 (−11.1, −5.1)	**<.001**	21.6 (18.7, 24.5)	15.9 (13.1, 18.7)	−5.7 (−8.7, −2.7)	**<.001**
Dyspnoea	12.8 (10.7, 15.0)	14.0 (11.9, 16.0)	1.2 (−1.7, 4.0)	.420	21.6 (18.4, 24.9)	18.5 (15.3, 21.6)	−3.2 (−6.5, 0.2)	.061
Insomnia	33.0 (29.9, 33.9)	31.0 (28.1, 33.9)	−2.0 (−5.9, 2.0)	.327	33.7 (29.7, 37.7)	28.4 (24.6, 32.2)	−5.3 (−9.4, −1.2)	.011
Appetite loss	14.1 (12.0, 16.3)	11.2 (9.1, 13.2)	−3.0 (−5.8, −0.2)	.036	8.7 (6.2, 11.2)	9.3 (7.0, 11.8)	0.7 (−1.9, 3.2)	.620
Constipation	13.2 (11.0, 15.5)	12.0 (9.9, 14.2)	−1.2 (−4.1, 1.7)	.419	11.2 (8.4, 14.0)	10.9 (8.2, 13.6)	−0.3 (−3.2, 2.6)	.850
Diarrhoea	7.5 (5.8, 9.2)	8.5 (6.8, 10.1)	1.0 (−1.3, 3.2)	.400	8.2 (5.9, 10.4)	7.1 (4.9, 9.2)	−1.1 (−3.4, 1.2)	.348
Financial difficulties	9.0 (6.7, 11.4)	10.0 (7.7, 12.2)	0.9 (−2.1, 4.0)	.560	10.4 (7.5, 13.4)	8.2 (5.4, 11.0)	−2.3 (−5.3, 0.8)	.141
**EORTC QLQ-BR23**								
**Function subscales/items**								
Body image	80.9 (78.6, 83.2)	81.4 (79.2, 83.6)	0.5 (−2.5, 3.5)	.759	77.4 (74.1, 80.8)	79.2 (75.9, 82.4)	1.7 (−1.7, 5.2)	.323
Future perspective	46.3 (43.2, 49.4)	50.0 (47.0, 53.0)	3.7 (−0.4, 7.8)	.078	56.0 (52.2, 59.7)	58.3 (54.6, 61.9)	2.3 (−1.6, 6.2)	.247
Sexual function	18.1 (15.9, 20.3)	15.3 (13.2, 17.4)	−2.8 (−5.6, −0.0)	.049	18.0 (15.0, 20.9)	19.2 (16.4, 22.1)	1.2 (−1.8, 4.3)	.423
Sexual enjoyment	60.5 (55.9, 65.2)	62.5 (57.8, 67.1)	1.9 (−4.3, 8.2)	.539	60.0 (53.7, 66.4)	61.4 (55.0, 67.7)	1.3 (−5.1, 7.7)	.686
**Symptom subscales/items**								
Systemic therapy side effects	15.3 (13.8, 16.8)	15.1 (13.7, 16.5)	−0.2 (−2.1, 1.7)	.846	21.1 (19.1, 23.0)	19.0 (17.1, 20.9)	−2.1 (−4.1, 0.0)	.047
Upset by hair loss	60.2 (49.9, 70.5)	54.2 (45.3, 63.2)	−5.9 (−18.4, 6.5)	.348	42.7 (31.1, 54.3)	49.8 (39.2, 60.3)	7.0 (−5.1, 19.2)	.255
Arm symptoms	25.0 (23.1, 26.9)	14.2 (12.4, 15.9)	−10.8 (−13.3, −8.4)	**<.001**	23.2 (20.7, 25.7)	11.0 (8.6, 13.4)	−12.2 (−14.8, −9.6)	**<.001**
Breast symptoms	30.4 (28.4, 32.3)	27.2 (25.3, 29.1)	−3.2 (−5.7, −0.7)	.014	22.9 (20.5, 25.2)	16.2 (13.9, 18.4)	−6.7 (−9.1, −4.3)	**<.001**

Adjusted means from participants who had responded to the questionnaire at least once. **Early postoperative measurement:** adjusted means for country. **1-year follow-up:** adjusted means for country and inclusion of axillary level 1 in CTV. *ANOVA F-test. *P* ≤ .0017 is considered statistically significant. **Global health and QoL:** the higher score the better HRQoL. **Function scales:** the higher score the better function. **Symptom scales:** the higher score the worse symptom.

### 3.2 Lymph-ICF

Results are presented both as adjusted mean scores and as proportions within the categories “no problem or a small problem” (score 0–24.99), “a moderate problem” (score 25–49.99), and “a severe or very severe problem” (score 50–100) [27].

As presented in Table 3a and Fig. 2, the SLNB only group reported significantly less dysfunction regarding Lymph-ICF total and on the domains physical function, mental function, and mobility activities at one-year follow-up.

**Table 3a:** Adjusted Lymph-ICF mean function scores (95% CI), at early postoperative measurement and at one-year follow-up in the SLNB + ALND versus the SLNB only group.

	Early postoperative measurement				1-year follow-up			
	SLNB + ALND (N = 457)	SLNB only (N = 503)	Mean difference	P*	SLNB + ALND (N = 475)	SLNB only (N = 501)	Mean difference	P*
**Lymph-ICF**								
Lymph-ICF total	21.7 (20.0, 23.4)	15.5 (13.8, 17.1)	−6.2 (−8.4, −4.0)	**<.001**	18.3 (16.3, 20.3)	11.5 (9.6, 13.4)	−6.8 (−8.8, −4.7)	**<.001**
Physical function	19.4 (17.6, 21.1)	10.9 (9.2, 12.5)	−8.5 (10.8, −6.2)	**<.001**	20.4 (18.0, 22.7)	9.1 (6.9, 11.4)	−11.2 (−13.7, −8.8)	**<.001**
Mental function	14.9 (12.9, 16.8)	9.8 (7.9, 11.7)	−5.1 (−7.6, −2.5)	**<.001**	13.6 (11.2, 16.0)	6.8 (4.5, 9.1)	−6.8 (−9.3, −4.4)	**<.001**
Household activities	20.6 (18.3, 22.8)	16.4 (14.2, 18.5)	−4.2 (−7.1, 1.3)	.004	17.1 (14.7, 19.6)	13.3 (10.8, 15.7)	−3.9 (−6.5, −1.3)	.003
Mobility activities	26.6 (24.4, 28.9)	20.8 (18.6, 22.9)	−5.8 (−8.8, −2.9)	**<.001**	21.2 (18.7, 23.6)	16.5 (14.1, 18.8)	−4.7 (−7.2, −2.2)	**<.001**
Life and social activities	25.1 (22.8, 27.5)	19.5 (17.3, 21.8)	−5.6 (−8.7, −2.5)	**<.001**	15.7 (13.3, 18.0)	12.1 (9.8, 14.4)	−3.5 (−6.0, −1.1)	.005

Adjusted means from participants who had responded to the questionnaire at least once. **Early postoperative measurement:** adjusted means for country. **1-year follow-up:** adjusted means for country and inclusion of axillary level 1 in CTV. *ANOVA F-test. *P* ≤ .0017 is considered statistically significant. **Lymph-ICF:** the higher score the more severe arm dysfunction.

The distribution of reported problems (Table 3b) in the above-mentioned categories was significantly in favour of the SLNB only group regarding Lymph-ICF total and on the domains physical function, mental function, mobility activities, and life and social activities at one-year follow-up. These differences were partly already seen at the early postoperative measurement: While arm dysfunction decreased in both randomization groups from the early postoperative measurement to one-year follow-up, this was not true for physical function which had increased dysfunction in the SLNB + ALND group at one-year follow-up (Table 3b).

**Table 3b:** Adjusted categorized Lymph-ICF arm dysfunction presented as crude frequencies and percentages and as odds ratio (95% CI) at early postoperative measurement and at one-year follow-up in the SLNB + ALND versus the SLNB only group.

	Early postoperative measurement				1-year follow-up			
	**SLNB + ALND N (%)**	**SLNB only N (%)**	**Odds ratio (95% CI)**	P*	**SLNB + ALND N (%)**	**SLNB only N (%)**	**Odds ratio (95% CI)**	P*
**Categorized Lymph-ICF**								
**Lymph-ICF total**								
No problem or a small problem	291 (65.1)	380 (78.0)	0.52 (0.39–0.69)	**<.001**	339 (72.9)	428 (87.0)	0.44 (0.31–0.62)	**<.001**
A moderate problem	112 (25.1)	84 (17.3)			97 (20.9)	48 (9.8)		
A severe problem or a very severe problem	44 (9.8)	23 (4.7)			29 (6.2)	16 (3.2)		
**Physical function**								
No problem or a small problem	317 (71.9)	413 (86.4)	0.39 (0.28–0.55)	**<.001**	308 (66.8)	426 (87.7)	0.29 (0.20–0.40)	**<.001**
A moderate problem	72 (16.3)	47 (9.8)			93 (20.2)	42 (8.6)		
A severe problem or a very severe problem	52 (11.8)	18 (3.8)			60 (13.0)	18 (3.7)		
**Mental function**								
No problem or a small problem	337 (77.1)	409 (86.6)	0.53 (0.38–0.75)	**<.001**	366 (79.6)	441 (91.5)	0.36 (0.24–0.54)	**<.001**
A moderate problem	59 (13.5)	32 (6.8)			52 (11.3)	21 (4.4)		
A severe problem or a very severe problem	41 (9.4)	31 (6.6)			42 (9.1)	20 (4.1)		
**Household activities**								
No problem or a small problem	305 (69.2)	365 (76.2)	0.69 (0.52–0.92)	.013	355 (77.0)	411 (84.9)	0.64 (0.46–0.91)	.012
A moderate problem	76 (17.2)	69 (14.4)			69 (15.0)	43 (8.9)		
A severe problem or a very severe problem	60 (13.6)	45 (9.4)			37 (8.0)	30 (6.2)		
**Mobility activities**								
No problem or a small problem	257 (57.6)	319 (65.9)	0.68 (0.53–0.88)	**.004**	305 (66.3)	371 (76.0)	0.66** (0.49–0.88)	**.005**
A moderate problem	99 (22.2)	101 (20.9)			108 (23.5)	78 (16.0)		
A severe problem or a very severe problem	90 (20.2)	64 (13.2)			47 (10.2)	39 (8.0)		
**Life and social activities**								
No problem or a small problem	274 (61.4)	335 (69.2)	0.71 (0.54–0.92)	.010	346 (75.2)	409 (84.0)	0.61 (0.44–0.86)	**.004**
A moderate problem	87 (19.5)	81 (16.7)			75 (16.3)	51 (10.5)		
A severe problem or a very severe problem	85 (19.1)	68 (14.1)			39 (8.5)	27 (5.5)		

Results from participants who had responded to the questionnaire at least once. **Early postoperative measurement:** odds ratio adjusted for country. **1-year follow-up:** odds ratio adjusted for country and inclusion of axillary level 1 in CTV. *Wald test. *P* ≤ .0083 is considered as statistically significant. **Proportional odds assumption was violated.

Since the proportional odds assumption was violated for the categorized outcome “mobility activities”, an additional nominal regression model was assessed. Patients in the SLNB only group had a 41% lower risk (RR: 0.59, 95% CI:0.42–0.83, *P* = .002) to have a “moderate problem” and a 21% lower risk (RR: 0.79, 95% CI:0.49–1.27, *P* = .32) to have a “severe or very severe problem” compared with the SLNB + ALND group.

## 4 Discussion

In the present analysis of the randomized SENOMAC trial, patient-reported arm symptoms and function one year after surgery were significantly better if completion ALND was omitted. HRQoL, however, was not affected by de-escalated axillary surgery.

The scarcity of published PROMs deriving from randomized trials assessing the impact of locoregional treatment on arm morbidity and HRQoL limits the possibility to compare our outcomes with other similar trials. The only detailed report stems from the ALMANAC trial published in 2006 [28] which states that ALND negatively affected PROMs when compared with SLNB alone in node-negative breast cancer.

In both the AMAROS and the OTOASOR trials, clinical assessment of lymphoedema and other arm symptoms was performed, and PROMs were also collected [7,8]. Despite of the significant impact of ALND on clinical signs of lymphoedema, (reported in 28% versus 15% and 15.3% versus 4.7% in the AMAROS and OTOASOR trials, respectively), PROMs did not show any significant differences between the randomization groups in either of the trials. Both trials observed that adding axillary RT to ALND further aggravated clinical signs of lymphoedema [7,8].

In contrast to the AMAROS and OTOASOR trials, the present analysis showed significant group differences on symptom subscales specific to the operated area. HRQoL, on the other hand, did not differ, probably because it may also be affected by other events in life [29], and in the context of breast cancer, by chemotherapy and endocrine therapy more than by surgery itself [19].

Both randomization groups reported a slightly better HRQoL compared with general Swedish and Danish population [30]. This observation may be explained by response shift, a normal adaption to a changed situation such as a disease or treatment-related symptoms. Response shift is suggested to have a positive impact on HRQoL [31].

### 4.1 Limitations

This analysis has several strengths. Firstly, data were collected in a prospective randomized setting with an intervention that was strictly controlled. Secondly, the excellent response rate renders representative results. Thirdly, even though the present analysis was carried out before full enrolment in the SENOMAC trial, this is one of the largest populations published on PROMs in the setting of de-escalation of axillary surgery.

One limitation of this analysis is the lack of a true baseline measurement, as explained in Materials and Methods section. While this may pose a difficulty in interpreting baseline data – here termed “early postoperative measurement” – it should not impact on the inter-group comparisons made after one year. However, since the SLNB + ALND group included participants with only SLNB but also with ALND at the time of the early postoperative measurement, its reported early postoperative PROMs may be worse than if all participants of this group had only undergone SLNB at that time.

Since objective measurements and PROMs not necessarily align [12,19,20], the lack of comparable objective measures in this analysis may limit the relevance of our results. This analysis reflects only the participants’ experiences at one-year follow-up between the randomization groups and should be considered from this perspective.

This analysis has no detailed information on received postoperative physiotherapy, which may have a positive effect on recovery of arm function [32]. Both Swedish and Danish sites routinely distribute at least written physiotherapy instructions, and in case of more extensive surgery, such as ALND or mastectomy, even individual physiotherapy is offered. Thus, obtained physiotherapy may have mitigated observed effects rather than enhanced them.

Finally, the risk that uneven distribution of unreported confounders, such as body mass index, physical activity, or socioeconomic status could have affected the outcome is deemed minimal due to the randomized trial design.

## 5 Conclusions

One year after surgery, arm function and symptoms, but not HRQoL, are significantly more impaired after completion ALND following SLNB than by SLNB only. These results are of high clinical relevance and underline the importance of integrating symptom-specific PROMs as well as overall HRQoL into the evaluation of de-escalation of axillary surgery.

## Declaration of competing interest

All listed authors declare that they have no conflict of interests.
