# Supplementary material for: Patient-reported outcomes one year after positive sentinel lymph node biopsy with or without axillary lymph node dissection in the randomized SENOMAC trial
Source: Breast. 2022 Mar 1;63:16–23. doi: 10.1016/j.breast.2022.02.013 (PMC8920917; doi:10.1016/j.breast.2022.02.013)
Supplement: Multimedia component 1 [file mmc1.docx]

Supplementary Table 1. Survey responders versus survey non-responders.

|  | **Survey responders**  **(N = 976)** | **Survey non-responders**  **(N = 205)** | ***P*** |  |
| --- | --- | --- | --- | --- |
|  |  |  |  |  |
| **Randomization group** |  |  | .399 |  |
| SLNB+ALND | 475 (48.7) | 93 (45.4) |  |  |
| SLNB only | 501 (51.3) | 112 (54.6) |  |  |
| **Type of breast surgery,** |  |  | .419 |  |
| BCS | 645 (66.1) | 129 (62.9) |  |  |
| Mastectomy | 331 (33.9) | 76 (37.1) |  |  |
| **Age,** median (range) | 62 (23-92) | 58 (37-94) | **.005** |  |
| <50 years | 175 (17.9) | 47 (22.9) | .053 |  |
| 50-65 years | 419 (42.9) | 95 (46.3) |  |  |
| >65 years | 382 (39.1) | 63 (30.7) |  |  |
| **Country** |  |  | .054 |  |
| Sweden | 733 (75.1) | 140 (68.3) |  |  |
| Denmark | 243 (24.9) | 65 (31.7) |  |  |
| **Chemotherapy*** |  |  | .329 |  |
| Yes | 658 (67.4) | 131 (63.9) |  |  |
| No | 318 (32.6) | 74 (36.1) |  |  |
| **Targeted therapy**** |  |  | .171 |  |
| Yes | 110 (11.3) | 16 (7.8) |  |  |
| No | 866 (88.7) | 189 (92.2) |  |  |
| **Endocrine therapy**** |  |  | .780 |  |
| Yes | 896 (91.9) | 187 (91.2) |  |  |
| No | 79 (8.1) | 18 (8.8) |  |  |
| Missing | 1 (0.1) |  |  |  |
| **Radiotherapy** |  |  | .290 |  |
| Breast/chest wall and regional lymph nodes | 918 (94.2) | 196 (95.6) |  |  |
| Breast/chest wall only | 34 (3.5) | 3 (1.5) |  |  |
| None | 23 (2.4) | 6 (2.9) |  |  |
| Missing | 1 (0.1) |  |  |  |

Presented as numbers and percentages if not stated otherwise. * Chemotherapy may be received before or after surgery. ** Ongoing treatment at one-year follow-up. SLNB: sentinel lymph node biopsy, ALND: axillary lymph node dissection, BCS: breast-conserving surgery.
